# Totally robotic repair of atrioventricular septal defect in the adult

**DOI:** 10.1186/s13019-015-0358-y

**Published:** 2015-11-06

**Authors:** Changqing Gao, Ming Yang, Cangsong Xiao, Huajun Zhang

**Affiliations:** Department of Cardiovascular Surgery, Institute of Cardiac Surgery, PLA General Hospital, 28 Fuxing Road, Beijing, 100853 China

**Keywords:** Atrioventricular septal defect, Mitral valve cleft, Robotics

## Abstract

**Background:**

Atrioventricular septal defect (AVSD) accounts for up to 3 % of congenital cardiac defects, which is routinely repaired via median sternotomy. Minimally invasive approach such as endoscopic or robotic assisted repair for AVSD has not been reported in the literature. With the experience with robotic mitral valve surgery and congenital defect repair, we initiated robotic AVSD repair in adults.

**Case Presentation:**

In this report, we presented three cases of successful repair of partial and intermediate AVSD by using da Vinci SI surgical system (Intuitive Surgical, Inc., Sunnyvale, CA).

**Conclusions:**

Totally robotic AVSD repair via right atriotomy could be safely performed in adults and it may provide superior cosmesis with the comparable surgical outcome of the repair via sternotomy.

## Background

Atrioventricular septal defect (AVSD) makes up to 3 % of all congenital cardiac defects, which is predominantly repaired via median sternotomy [1]. Totally robotic repair of AVSD has not been reported in the literature to our knowledge. We describe our experience with three cases of atrioventricular septal defect repair by using da Vinci SI surgical system (Intuitive Surgical, Inc., Sunnyvale, CA).

## Case presentation

Patient 1 A 33 year-old lady who had detected heart murmur for 6 years presented with recent onset of palpitation and chest discomfort. She was admitted in July 2011 and trans-thoracic echocardiography (TTE) suggested an ostium primum atrial septal defect (ASD) (2.2 cm in diameter) with left to right intracardiac shunt, and a 0.2 cm mitral valve cleft with moderate mitral regurgitation (MR). The interventricular septum was intact. The pulmonary artery pressure was 38 mmHg and mild tricuspid regurgitation was present. She was diagnosed with partial AVSD.

Patient 2 A 24 year-old lady was admitted due to detected heart murmur for 5 years in December 2014. TTE suggested an ostium primum ASD (1.2 cm) with mitral valve cleft and moderate MR whilst a patent foramen ovale (PFO) (0.2 cm) and a perimembranous ventricular septal defect (VSD) (0.3 cm) were also present with left to right shunt. The left atrium was dilated (3.6 cm in diameter). The ventricles were normal with LVEF of 76 %. She was diagnosed with intermediate AVSD and PFO.

Patient 3 A 27 year-old lady was admitted because of heart murmur detected 4 months before in January 2015. TTE suggested a sucundum ASD (2 cm) co-existing with an ostia primum ASD (2 cm) and a 1.1 cm mitral valve cleft, which caused left to right shunt and moderate MR. The pulmonary artery pressure was 41 mmHg and mild tricuspid regurgitation was seen. The right heart was dilated and the diameters of RA and RV were 4.6 cm and 4.3 cm respectively. The left heart was normal and LVEF was 58 %. The diagnosis was partial AVSD and sucundum ASD.

All three patients had normal peripheral vasculatures confirmed by ultrasonic scan and the informed consent was taken for totally robotic repair of AVSD before surgery.

**Fig. 1:**
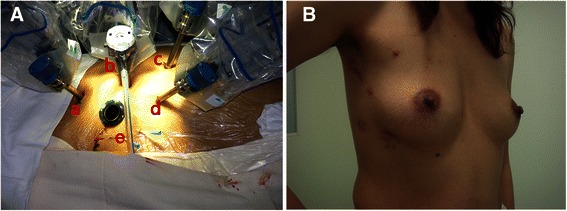
**a** The set-up of robotic camera (*b*) and instrument arms (*a*, *c*, *d*) in the right chest of patient, with a 1.5 cm working port (*e*) at the fourth intercostal space. **b** The surgical incision wounds on the chest wall at 6-month follow-up of patient 1

During surgery, general anaesthesia was induced and CPB was set up through peripheral vessels according to our protocol [2, 3]. After systemic heparinization, femoral arterial (17 F to 22 F) and venous (21 F or 23 F) cannulation (Metronic, Minneapolis, Minn) was performed through a 2-cm transverse incision at the right groin under the guidance of TEE. Bicaval venous drainage was instituted through the jugular and femoral/inferior vena cava cannulas. The robotic camera and instrument arms were inserted with a working port made in the right chest (Fig. 1). The pericardium was opened and the aorta was cross clamped after CPB began. The heart was arrested with single dose of antegrade cold HTK cardioplegic solution given directly through anterior chest using a 14GA angiocatheter, and endoscopic snaring of the vena cavae with umbilical cords was performed.

**Fig. 2:**
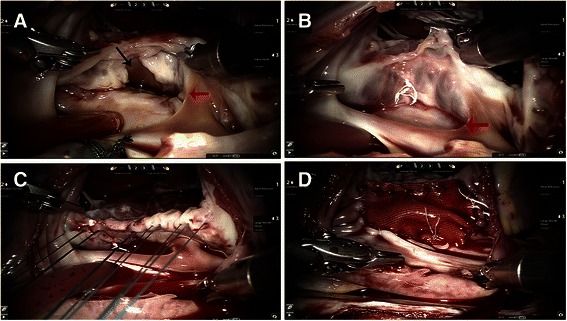
**a** A cleft between the left superior and inferior leaflets (*black arrow*) was examined and repaired through the osmium primum ASD (*red arrow*). **b** The saline injection showed a competent mitral valve after the repair by interrupted 4–0 Gore-Tex sutures. **c** The interrupted mattress sutures were placed along the crest of the ventricular septum with the pledgets on the right ventricular aspect. **d** The primum ASD was closed with a Dacron patch with coronary sinus draining to left atrium

Via right atriotomy, mitral valve was examined (Fig. 2a) and the cleft between the left superior and inferior leaflets was repaired using three to four interrupted 4–0 Goretex sutures and saline injection test was performed (Fig. 2b). Six to seven interrupted 4–0 pledgted Gore-Tex mattress sutures were placed along the crest of interventricular septum from the right ventricular side (Fig. 2c). In one case where a small VSD co-existed, the mattress sutures were placed beneath the lower margin of the VSD and brought directly through the crest of ventricular septum for the primary closure of VSD. After measurement of the dimension between the superior and inferior commissures was made, a Dacron patch was prepared according to the shape and size of the ostium primum ASD. Then, the mattress sutures were brought through the lower margin of the patch to secure it into the crest of ventricular septum, either by knot tying pusher or Cor-Knot™ tying device (LSI Solutions, Victor, NY). Then two 4–0 Gore-Tex running sutures were used to close the ASD from the upper and lower commissures. The inferior suture was continued below the Thebesian valve to avoid injury to the conduction tissue with the coronary sinus draining into the left atrium. PFO or secundum ASD was closed separately. The right atriotomy was closed with double layers of 4–0 Goretex running suture and CPB was weaned as routine.

**Fig. 3:**
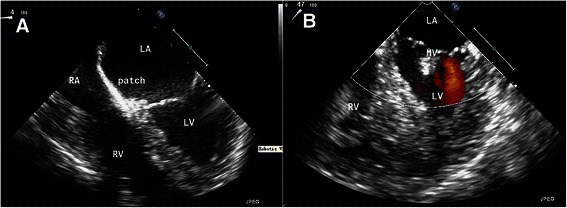
The postoperative TEE confirmed an intact septum without residual shunt (**a**) and competent mitral valve without regurgitation (**b**) after surgery

All three patients were off bypass easily. The total CPB time was 195 min, 128 min, and 126 min for the first, second and the third patients, and aorta cross clamping time was 126 min, 88 min and 80 min respectively. Intraoperative TEE excluded residual shunt and regurgitation of mitral or tricuspid valves. The patients were ventilated for 15 to 16 h in critical care ward and they all had an uneventful recovery postoperatively. Postoperative TEE confirmed that the robotic repair of the defects was successful with no intracardiac shunt or MR (Fig. 3). All patients were discharged within 1 week of surgery.

## Discussion

Unlike sucundum ASD and perimembranous VSD that can sometimes be occluded directly through the interventional procedure, AVSD is a more complex congenital heart defect and surgical repair is inevitable [1]. AVSD is conventionally repaired through median sternotomy, but no result on robotic repair of AVSD has been reported. Since most young adult patients are uneasy about the surgical wound, minimally invasive surgery is often sought by them. Although robotic cardiac surgery remains controversial due to steep learning curves, high expenses, long operation time, and highly selected patients, it has been developed over the last decade and is shown to improve cosmesis and quality of life after surgery [4]. We initiated the surgical repair of AVSD in 2011 and completed three cases so far.

Before robotic surgery, surgical approach had to be carefully planned to enable sufficient exposure and effective repair of the defects. Since right atriotomy through the right chest had provided us with excellent and reliable exposure of the right heart and the left atrium for robotic repair of ASD [3], VSD [2, 5], mitral valve [6], atrial mass [7, 8], and PS (data not published), the initial attempt of novel robotic repair of AVSD via right atriotomy was made with success. The long operation time was thought to be a shortcoming of robotic surgery. With robotic surgical experience gained, the operative duration could be significantly shortened. The aorta cross-clamping time decreased from 128 min for the first case of AVSD to around 80 min after performing a variety of robotic cardiac operations over a 3-year period.

The robotic surgical system has other advantages in the repair of AVSD. The endoscopic system provides the surgeon with excellent visualization of the delicate structure of the crest of septum and the surrounding valvular tissue with magnification higher than 10 times; hence, the injury to the cardiac conduction system could be effectively avoided. Besides, the surgical videos recorded is excellent media for surgical education.

## Conclusions

Totally robotic AVSD repair via right atriotomy could be safely performed in adults by an experienced robotic cardiac surgeon. The robotic repair technique may provide superior cosmesis with the comparable surgical outcome of conventional AVSD repair via median sternotomy.

### Consent

Written consent which had been approved by the institutional review board was taken from the patient for publication of this work and the associated images.

## Abbreviations

ASD: Atrial septal defect
AVSD: Atrioventricular septal defect
CPB: Cardiopulmonary bypass
MR: Mitral regurgitation
PFO: Patent foramen ovale
RA: Right atrium
TEE: Transesophageal echocardiography
TTE: Transthoracic echocardiography
VSD: Ventricular septal defect
